# The Second Face of Blindness: Processing Speed Deficits in the Intact Visual Field after Pre- and Post-Chiasmatic Lesions

**DOI:** 10.1371/journal.pone.0063700

**Published:** 2013-05-08

**Authors:** Michał Bola, Carolin Gall, Bernhard A. Sabel

**Affiliations:** Institute of Medical Psychology, Medical Faculty, Otto-von-Guericke University of Magdeburg, Magdeburg, Germany; University of Bologna, Italy

## Abstract

**Purpose:**

Damage along the visual pathway results in a visual field defect (scotoma), which retinotopically corresponds to the damaged neural tissue. Other parts of the visual field, processed by the uninjured tissue, are considered to be intact. However, perceptual deficits have been observed in the “intact” visual field, but these functional impairments are poorly understood. We now studied temporal processing deficits in the intact visual field of patients with either pre- or post-chiasmatic lesions to better understand the functional consequences of partial blindness.

**Methods:**

Patients with pre- (n = 53) or post- chiasmatic lesions (n = 98) were tested with high resolution perimetry – a method used to map visual fields with supra-threshold light stimuli. Reaction time of detections in the intact visual field was then analyzed as an indicator of processing speed and correlated with features of the visual field defect.

**Results:**

Patients from both groups exhibited processing speed deficits in their presumably “intact” field as indicated by comparison to a normative sample. Further, in both groups processing speed was found to be a function of two factors. Firstly, a spatially restricted (retinotopic) influence of the scotoma was seen in longer reaction times when stimuli were presented in intact field sectors close to the defect. Secondly, patients with larger scotomata had on average longer reaction times in their intact field indicating a more general (non-retinotopic) influence of the scotoma.

**Conclusions:**

Processing speed deficits in the “intact” visual field of patients with visual system damage demonstrate that visual system lesions have more widespread consequences on perception than previously thought. Because dysfunctions of the seeing field are expected to contribute to subjective vision, including visual tests of the presumed “intact” field may help to better understand vision loss and to improve methods of vision restoration and rehabilitation.

## Introduction

**Figure 1:**
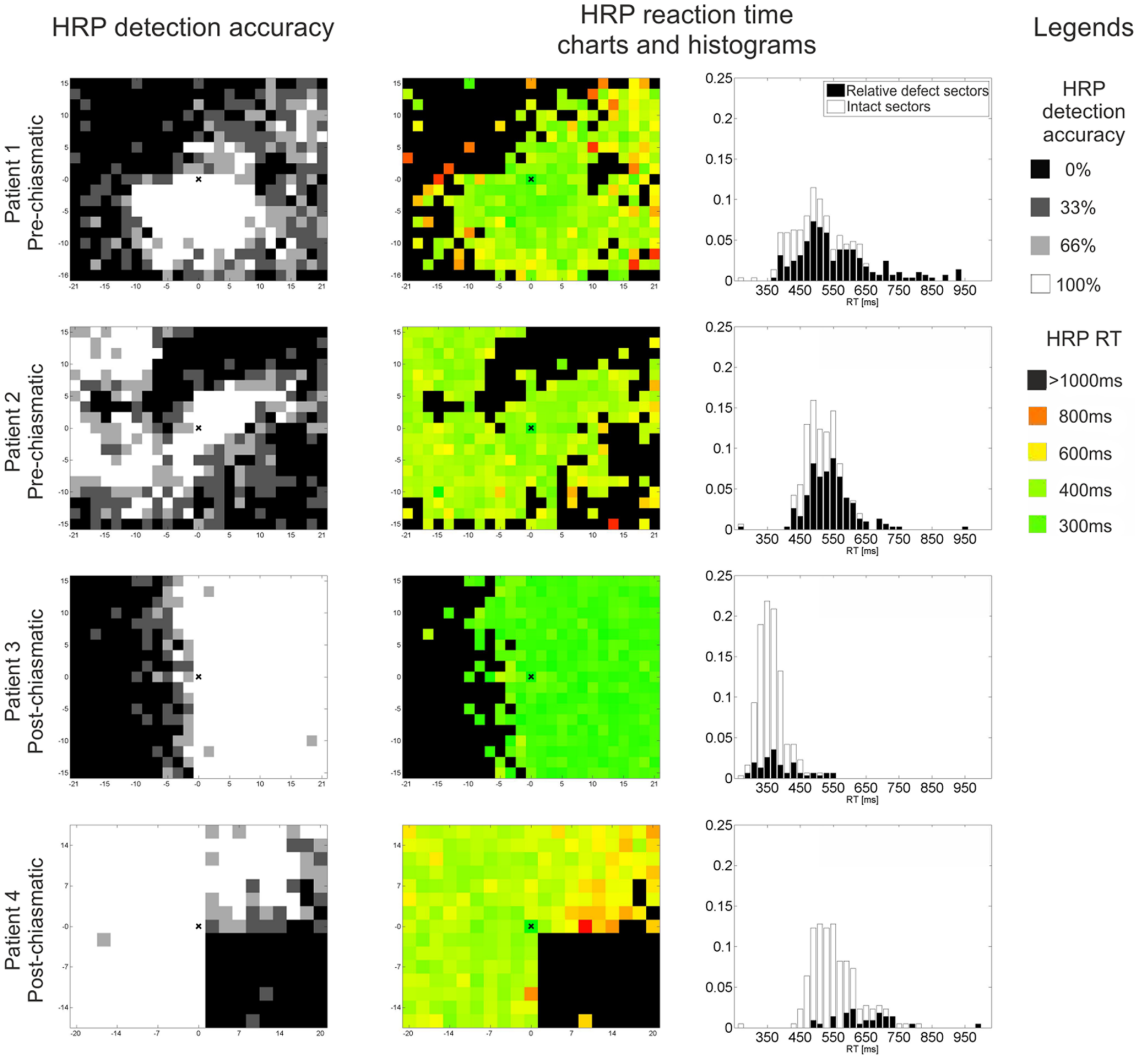
Visual field charts of patients with pre-chiasmatic and post-chiasmatic visual system damage. Detection accuracy of stimuli presented in high resolution perimetry (HRP), an indicator of functional state (defect depth), is denoted by color: black represents absolute defect (0% detection), grey represents areas of residual vision (relative defect; detection >0% and <100%) and white represents intact field (100% detection). Reaction time (RT) is color-coded in RT charts. RT histograms show the distribution of RT in the HRP sectors defined as intact or relative defect.

When structures along the cerebral visual pathway are damaged, vision is lost in sectors of the visual field that retinotopically correspond to the damaged tissue. The blind region, called scotoma or blind field, varies in localization and size depending on the locus and extent of the lesion [1], [2]. Scotomata are the result of different diseases such as glaucoma, optic nerve damage, or hemianopia following posterior artery stroke. Such visual field loss is typically tested with perimetry assessing the detection accuracy of near-threshold or supra-threshold stimuli across the visual field [3]. Data collected in this manner are then used to construct visual field charts (**Fig. 1**) showing defective (blind) regions which can be subdivided into areas of relative defect (also called “areas of residual vision”, ARV, [4]), and areas of absolute blindness. As the remaining sectors of the visual field are considered fully intact, presumably without any visual dysfunction, it is believed that the scotoma is the only vision problem that patients encounter.

Thus, in clinical work, and in most research studies, there are two general assumptions: (i) perception is completely abolished in the “blind” field and (ii) perceptual functions are “normal” in the intact field. But neither assumption seems true. With regard to the former, the discovery of the “blindsight” phenomenon in 1973 shows that visual information is still processed deep in the “blind” field, allowing patients to correctly detect the presence of stimuli without being aware of them [5]–[9]. With regard to the latter assumption, there is already some evidence that the presumably “intact field” has actually some subtle perceptual deficits which remain undetected by static perimetry.

The intact field of patients with unilateral visual cortex lesions shows decreased contrast sensitivity [10] and an impaired ability to detect targets on a noisy background [11] when compared to control subjects without brain damage. Deficits of higher perceptual functions were also found such as impaired figure-ground segregation [12], [13] and maladaptive patterns of visual field search [14], [15]. Importantly, in all studies that compared reaction time (RT) between patients and control subjects, regardless of whether simple [16], [11], [17] or more complex stimuli were used [12], [13], [18], RT was found to be significantly longer in patients. Therefore, an impaired visual processing speed stands out as a main feature of the “intact” field.

Further, not only cortically lesioned patients experience perceptual problems in the seeing field but also subjects with damage to the optic nerve (ON) due to optic neuritis. Despite exhibiting normal detection abilities in their visual field, patients with optic neuritis are impaired in motion detection and temporal processing tasks [19]–[21]. Longitudinal studies showed that perception of stationary stimuli, as presented in perimetry, recovers fast after the optic neuritis episode, but deficits in dynamical aspects of perception persist [20], [21]. This was related to reduced activity in the motion processing area MT+/V5, as shown by fMRI [20], and to longer latencies of visual evoked potentials [21].

Visual field loss resulting from lesions to the visual system occurring at different levels, from the retina to the primary and extrastriate visual cortex, severely affects vision-related quality of life [22], [23]. Perceptual deficits, if they occur in the remaining, seeing field, are expected to hamper subjective quality of vision even further. However, these are neither diagnosed nor properly understood. Apparently, we have turned a “blind eye” on the seeing field dysfunctions, consequently underestimating the extent of visual impairment in partially blind patients.

In the present study we analyzed data from patients with pre-chiasmatic and post-chiasmatic visual system damage, suffering from chronic visual field loss. Both groups performed the same visual detection task, and both exhibited intact field processing speed deficits as indicated by comparisons to uninjured, healthy controls. As not much is known about the mechanisms of intact field deficits, the main aim of the present study was to define features of the visual field defect related to the degree of the deficit. It is likely that structural damage on the border of the lesion or disrupted short-range interactions may account for the intact field perceptual problems. Therefore, processing speed should be more severely impaired in the vicinity of the defect, proving that the scotoma has a local (retinotopic) effect on the adjacent intact field. At the same time, firstly, dense visual field testing shows that perceptual deficits are not restricted to the immediate vicinity (or border) of the lesion [11], [16], and secondly, patients are impaired also in tasks requiring long-distance spatial integration of information [12], [13], [20], [21]. Both observations suggest that the scotoma might also have a non-specific, global effect, which is expected to affect the whole visual field in a non-retinotopic manner.

As we now show, intact field processing speed is impaired in both groups, pre- and post-chiasmatic, when compared to uninjured, control subjects. Further, in both groups two factors were associated with the speed of processing: (i) RT was longer when a stimulus was presented close to the visual field defect and (ii) patients with larger scotomata had longer RT in their intact field. Thus, the former indicates a local (retinotopic) effect of the scotoma on processing visual information within the intact visual field, while the latter indicates a global (non-retinotopic) effect on the whole visual field.

## Methods

### Ethics statement

This study was conducted according to the Declaration of Helsinki and it was approved by the ethics committee of the Otto von Guericke University of Magdeburg. Written informed consent was obtained from all participants prior to the experiments.

### Subjects

We retrospectively analyzed data on patients' visual fields using a database consisting of 53 patients with pre-chiasmatic and 98 patients with post-chiasmatic damage. To be included subjects had to meet the following criteria: (i) chronic visual system damage (>6 months after damage); (ii) presence of residual vision as detected by high resolution perimetry (HRP); (iii) sufficient fixation ability; and (iv) compliance with the experimenters' instructions. Based on additional exclusion criteria (see below) we excluded 13 pre- and 6 post-chiasmatic subjects. The analyzed groups (pre-chiasmatic, n = 42; post-chiasmatic, n = 92) were matched according to age and gender (see: **Table 1**).

**Table 1:** Sample descriptions of the pre- and post-chiasmatic group.

	Pre-chiasmatic	Post-chiasmatic	p
*N*	42	92	
Gender (F/M)	15/27	32/60	0.91
Age (years)	54.9	56.10	0.71
Fixation accuracy (%)	97±0.004	96±0.002	0.19
False positive rate (%)	0.82±0.18	0.38±0.04	0.06
Intact field (%)	48±4	58.4±1	0.007
Relative defect (%)	21.8±2	7±0.5	<0.001
Absolute defect (%)	30±4	34.5±1	0.21
Diffuseness (%)	52.1±4	19.5±1	<0.001

Groups did not differ significantly in age, fixation accuracy, and proportion of the absolute visual field defect. However, visual fields in the pre-chiasmatic sample were overall more diffuse, as indicated by a significantly higher proportion of relative defects which was accompanied by a lower proportion of the intact field.

Concerning the etiology, most patients in the post-chiasmatic group suffered from stroke (n = 83) and a minority from trauma (n = 5) and tumor (n = 4). In the pre-chiasmatic group etiologies were more variable, including: arteritic anterior ischemic optic neuropathy (AION, n = 13), ON inflammation (n = 7), idiopathic ON atrophy (n = 6), tumor (n = 5), retinal artery occlusion (n = 4), non-arteritic anterior ischemic optic neuropathy (NAION, n = 2), glaucoma (n = 2), trauma (n = 2), ON atrophy in Bechterews disease (n = 1).

We compared the processing speed of both patients groups to a normative sample from the Tölz Temporal Topography study which used the same methodology of visual field testing [24]. The mean age of pre- and post-chiasmatic subjects was 54.9 yrs. and 56.1 yrs., respectively. The age of the normative data group was 50–59 yrs. (n = 14; 8 female). Both patient groups were gender-matched to the normative group (chi-square(1,54) = 1.99, p = 0.158; chi-square(1,10) = 2.58, p = 0.108).

### Clinical examination

All patients were tested with high resolution perimetry (HRP). We chose to reanalyze HRP data as this method has been used for many years in our laboratory in different studies which provided the database for our study. Moreover, HRP provides an excellent spatial resolution and enables reaction time recording and analysis. Details of HRP are described elsewhere [16], [24]–[27].

Briefly, HRP measurements were conducted in a darkened room, where patients were seated in front of a 17" monitor with a chin-rest and forehead-holder. In this manner the eyes were kept at a constant distance of 42 cm from the screen. Supra-threshold, white light stimuli were presented continuously on dark grey background, within a grid of 25x19 or 19x15 sectors, covering ±20° horizontally and ±15° vertically of subjects' visual field. In each block one stimulus per sector was presented. The order of stimulus positions and the length of inter-stimulus intervals (ISI; from 1000 to 2000ms) were random so that patients could not predict when and where the stimuli were presented. A fixation point placed in the middle of the screen served as reference to set up the screen in the optimal height (keeping the patients' eyes at the level of the fixation point). In order to control subjects fixation, during the testing an isoluminant color change of the fixation point was presented 80 times per block. Subjects were instructed to maintain fixation at all times and press the space bar on the keyboard whenever a light stimulus was detected or a color change of the fixation point (fixation control) occurred. At 1000ms after presentation of a light stimulus or fixation control the next trial started, beginning with a random ISI, followed by a stimulus presentation or another fixation control. RT values faster than 150ms and slower than 1000ms were treated as “false positives”. These cut-offs were used as this allows sufficient time to respond to the stimuli (see: histograms in **Fig. 1**) and effectively prevents guessing. RT upon “hits” were saved and analysed as an indicator of visual processing speed.

Detection accuracy in every sector averaged over blocks of three repetitions was used as a criterion to define whether a given sector of the visual field belonged to the “intact” area (100% stimuli detected, shown in white in the visual field charts, **Fig. 1**), mild relative defect area (66% stimuli detected, light grey), moderate relative defect (33% stimuli detected, dark grey) or absolute defect area (0% stimuli detected, black).

### Exclusion criteria

Detection accuracy of the color change at fixation was taken as an indicator of fixation stability. We excluded subjects with fixation accuracy below 90% which resulted in the exclusion of 7 pre-chiasmatic subjects (7/53) and no subjects (0/98) from the post-chiasmatic group.

Additionally, we conducted all analyses (data not shown) also with a more strict fixation criterion by excluding subjects with fixation accuracy < 95%. This resulted in the exclusion of 15 subjects (15/53) from the pre-chiasmatic group and 28 subjects (28/98) from the post-chiasmatic group. This analysis led to essentially similar results than those of the more liberal exclusion as described below. However, the stricter fixation criterion reduced the strength of correlations and decreased variability among subjects.

Furthermore, we excluded subjects with false positive rates >5% which resulted in exclusion of four subjects (4/46) from the pre-chiasmatic and six subjects (6/98) from the post-chiasmatic group. After applying all exclusion criteria the final sample to be analyzed consisted of 42 pre-chiasmatic subjects and 92 post-chiasmatic subjects (see: **Table 1**).

### Visual field characteristics

For every subject the size of each visual field functional “state” (intact, relative defect, absolute defect) was determined and expressed as percentage of HRP sectors belonging to each state. Both analyzed groups (**Table 1**) did not differ with respect to the extent of the absolute defect areas (t(1,132) = 1.25, p = 0.21), fixation accuracy (t(1,132) = 1.31, p = 0.19), and false positive rate (t(1,132) = 1.89, p = 0.06). However, post-chiasmatic patients had significantly larger intact areas (t(1,132) = 2.73, p = 0.007), and smaller relative defect areas (t(1,132) = 3.56, p<0.001). Thus, both groups differed with respect to the structure of the visual field defect: while pre-chiasmatic lesions typically led to more diffuse defects with broader transition zones (relative defects), post-chiasmatic subjects are characterized by a higher probability of clear-cut scotoma borders (**Fig. 1**). To more explicitly quantify the “diffuseness” of the scotoma we calculated the proportion of relative defect sectors in the scotoma by using the formula:

*relative defect size/(relative defect size + absolute defect size)*

This indicator was also significantly different between groups (t(1,132) = 3.82, p<0.001).

### Influence of scotoma features on intact field RT

The main aim of the study was to define factors associated with processing speed in the patients' intact field. To this end we firstly studied the topography of RT, namely whether processing speed is similar across the whole intact field, or if there are some regions which are more deficient than others. We hypothesized that RT might be slower especially in areas adjacent to the scotoma because the local, lateral interactions are disturbed. The most straightforward approach to test this hypothesis was to define the lesion border of each patient and then analyze intact field RT as a function of the distance to the border. However, due to a high variability of visual field topography, especially in the pre-chiasmatic group (**Fig. 1**), it was difficult to objectively define the scotoma border position. Therefore, we decided to analyze RT of intact sectors as a function of the number of defective sectors located within a 5-degree surround because this size approximates the receptive field size in the human early visual cortex [28]. Because in this analysis we were not interested in interhemispheric effects, only spots positioned on the same side of the vertical meridian were considered. All intact sectors for each particular subject were then divided into four categories: (i) 0% defective sectors in the surround; (ii) 1%–25% defective sectors in the surround; (iii) 26%–50% defective sectors in the surround; (iv) more than 50% of defective sectors in the surround. Then, the average RT for each category was subjected to statistical analysis.

Secondly, to study global (non-retinotopic) effects of the scotoma, we conducted correlations between RT and scotoma size. We hypothesized that subjects with larger scotomata might be more impaired in intact field processing. To address this issue the size of the defective visual field was correlated with the averaged RT in the intact visual field. Because central sectors of the visual field occupy a larger cortical representation than peripheral regions, the HRP visual field charts were transformed according to the cortical magnification factor (CMF) as defined by Qiu et al. [29]. This was done to avoid over- or underestimating the functional impact of defects in the periphery versus the central visual field. However, when comparing correlations involving the CMF-corrected versus non-corrected proportion of the defect field, it was noted that the CMF correction had only a minor effect on our study results.

Thirdly, to study interhemispheric influences, a sub-population of post-chiasmatic patients was studied that suffered a loss of vision in only one quadrant (n = 16; 8 female). The mean age in this subgroup was 52±4 yrs. and the results were compared to the normative sample with an age-range of 50–59 yrs. (n = 14; 8 female). Groups were gender-matched (chi-square(1,28) = 0.153, p = 0.696). This subgroup analysis allowed to investigate RT within three “intact” quadrants that are subject to different kinds of influences depending on their location and distance in relation to the scotoma: (i) the adjacent quadrant (lower or upper) in the same hemisphere which is possibly influenced by intrahemispheric interactions; (ii) the adjacent quadrant in the contralateral hemisphere which is possibly influenced by the loss of interhemispheric connections caused by the lesion in the mirror-symmetric position; and finally (iii) the diagonal quadrant in the contralateral hemisphere that we expected to be influenced the least by the lesion.

### Statistics

To compare the pre- and post-chiasmatic patient groups with normative data from the Tölz Temporal Topography Study [24] a t-test for independent samples was applied. To compare gender distribution chi-square test was used. To investigate effects of visual field features on RT we used repeated measures ANOVA with within-subject factor being the visual field feature. We tested three visual field features: detection accuracy (3 levels), accuracy in the surround (4 levels), and visual field quadrant (3 levels). Post-hoc tests were Bonferroni corrected. The relationship between scotoma size and RT was analyzed with Spearman correlation coefficient as this test is more robust to outliers than the Pearson coefficient [30].

Values are presented as mean ± standard error of the mean (SEM). The two-tailed significance criterion was set at p<0.05. Greenhouse-Geisser correction was applied when necessary. All analyses were carried out in Matlab 2011b (Mathworks) and SPSS 21.

## Results

### Intact field RT is higher in patients than in control subjects

Firstly, we compared intact field RT of both patients groups, pre- and post-chiasmatic, to normative data of healthy subjects (347.2±4ms) in the Tölz Temporal Topography Study [24]. In both, pre-chiasmatic (469.4±10ms; t(1,41) = 12.18, p<0.001) and post-chiasmatic patients (437.4±6ms; t(1,91) =  13.8, p<0.001) RT was significantly longer than in the control group.

Further, intact field RT of patients was compared to control group, but including only RT of intact sectors with completely intact 5 deg. surround. Still, both groups of patients, pre-chiasmatic (439.8ms, t(1,35) = 10.62, p<0.001) and post-chiasmatic (429.7ms, t(1,91) = 14.61, p<0.001), exhibited longer RT than control subjects.

### RT is related to the functional state of the visual field

**Figure 2:**
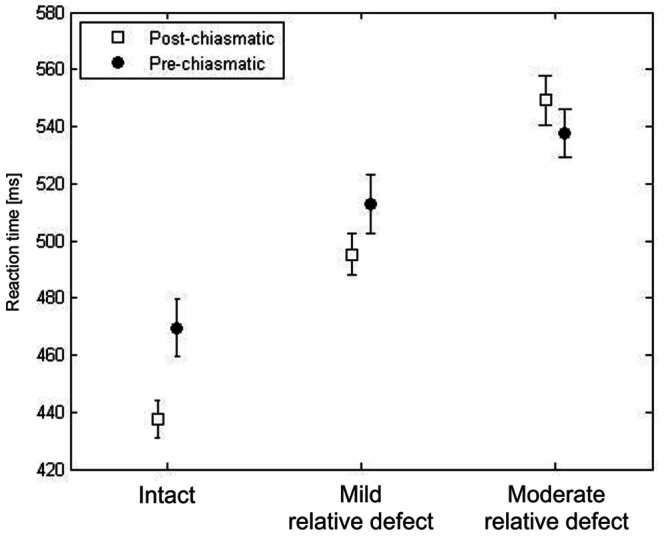
Reaction time is related to the functional state of the visual field area. In both groups, pre-chiasmatic and post-chiasmatic, RT depends on the functional state of the visual field sector, as defined by HRP detection accuracy. Processing speed upon detected stimuli was higher in the relative defect regions than in the intact field.

In both patient groups RT was found to be related to the visual field functional state, i.e. defect depth (**Fig. 2**, pre-chiasmatic: F(2,39) = 84.02, p<0.001; post-chiasmatic: F(2,94) = 134.24, p<0.001). All post-hoc comparisons between three visual field states were significant with p<0.001.

### Local effect: intact field RT is related to the functional state of the immediate surround

**Figure 3:**
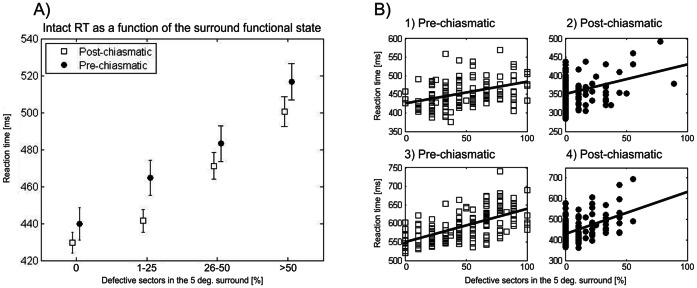
Intact field reaction time is related to the functional state of the immediate surround. Group analysis (A) and single cases as representative examples (B) are shown. Functional status of the immediate neighborhood, defined as the percent of defective sectors in the 5-degree surround, partially explains within-subject variability of RT in patients with pre-chiasmatic (case 1 and 3) and post-chiasmatic lesions (case 2 and 4). However, between-subject RT variability is still substantial.

RT at any tested position in the intact visual field depends on the functional state of its immediate surround (**Fig. 3**; pre-chiasmatic: F(3,29) = 77.24, p<0.001; post-chiasmatic: F(3,73) = 68.47, p<0.001). All post-hoc comparisons between intact sectors with different surround states were significant (p<0.001). This shows that processing speed deficits are not uniformly distributed but intact visual field regions are more impaired when located in the vicinity of the scotoma.

Scatterplots of single cases (**Fig. 3** **, B**) show the association between RT of a particular HRP sector and the proportion of defective sectors in its surround. However, the inter-subject variability of RT is still rather high suggesting that additional, global factors have an impact on processing speed in the intact visual field.

### Global effect: intact field RT is related to the scotoma size

**Figure 4:**
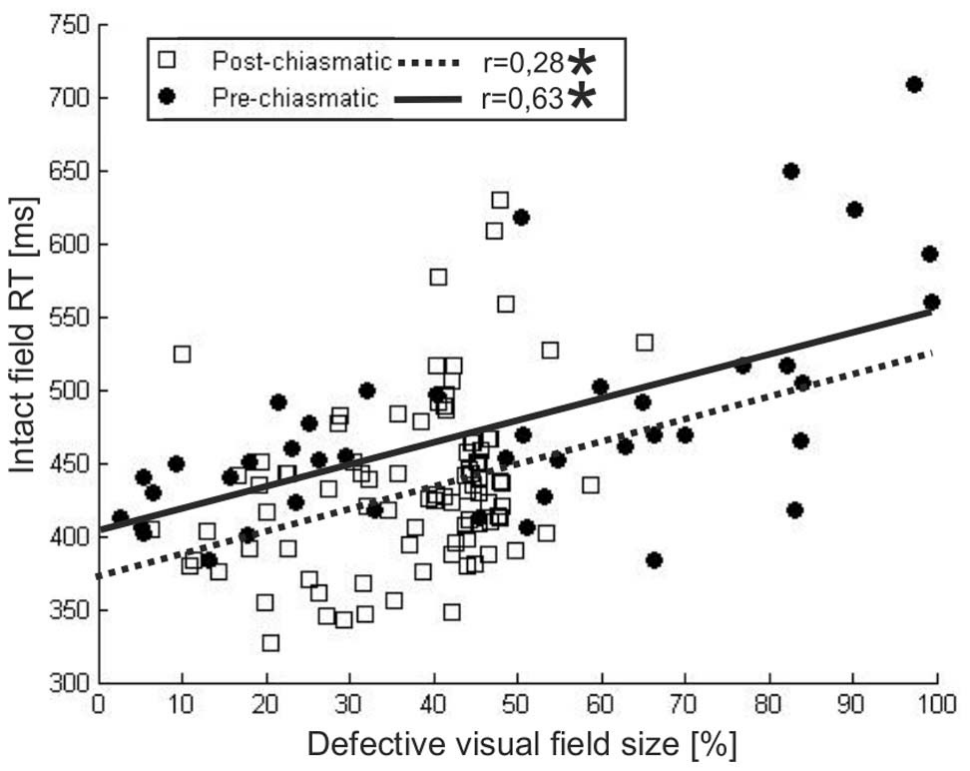
Intact field reaction time is related to the size of the scotoma. In both groups, pre-chiasmatic and post-chiasmatic, a significant correlation was found between the size of the scotoma and the intact field RT. This indicates that subjects with a larger defect field are more impaired in the intact field as well.

Larger defective visual fields were associated with longer RT averaged over the whole intact field in both groups, pre-chiasmatic (r = 0.63, p<0.001) and post-chiasmatic (r =  0.28, p = 0.006) (**Fig. 4**). These correlations were also significant when only the absolute defect area (proportion of black sectors only) was considered (pre-chiasmatic: r = 0.53, p = 0.001; post-chiasmatic: r = 0.23, p = 0.022). However, it might be argued that the “local effect” contributes mainly to these correlations as the mere presence of a larger defective field implies a greater proportion of the intact field located in the vicinity of the defect. To refute this possibility the RT of all intact sectors were z-normalized for the proportion of defective sectors in the 5-degree surround. In line with our hypothesis, correlations with normalized RT were weaker but still significant for pre-chiasmatic (r = 0.35, p = 0.022) and post-chiasmatic patients (r = 0.27, p = 0.008), which proves that a local influence alone cannot explain longer RT in patients with greater defect size.

### Interhemispheric effect: intact field RT in quadrantanopia

**Figure 5:**
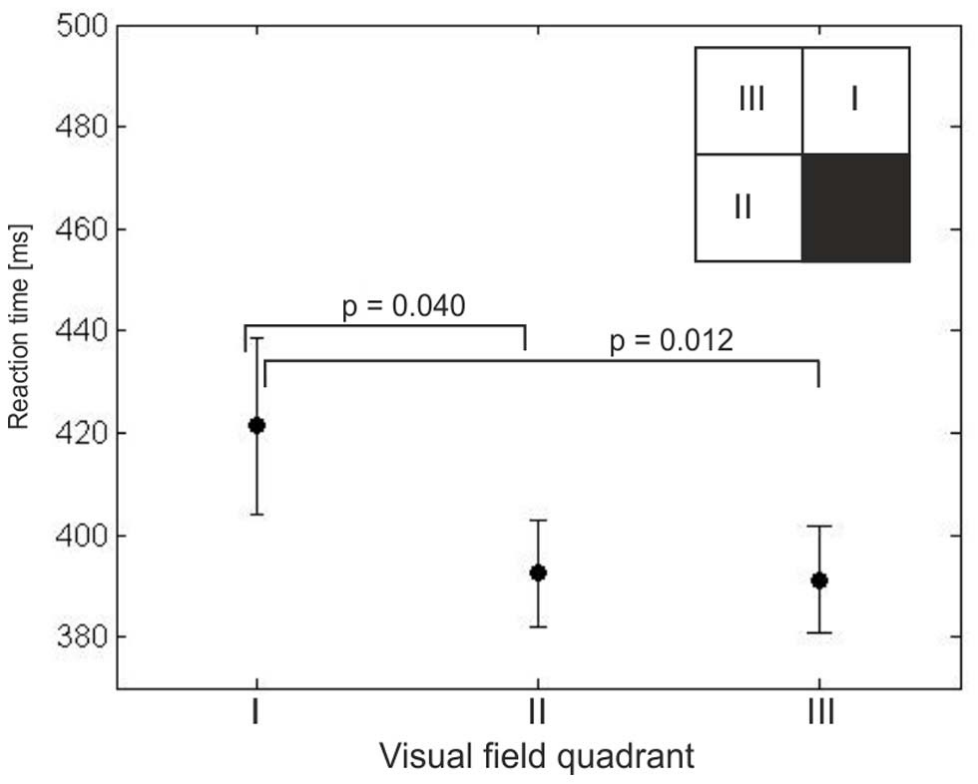
Intact field reaction time in quadrantanopia. RT was calculated for each of the three quadrants with respect to the position of each quadrant in relation to the scotoma: RT in the adjacent quadrant of the same hemisphere (I) was slower than in the adjacent (mirror-symmetric) quadrant of the contralateral hemisphere (II) and the diagonal quadrant (III). The inset is a simplified sketch of a visual field test showing an example of a lower right quadrantanopia to illustrate the position of the three “intact” quadrants. Processing speed in all three intact quadrants was impaired in comparison to healthy controls. Additionally, the intact quadrant located in the same hemispace as the scotoma (I) was found to be more impaired than the two quadrants processed by the uninjured hemisphere (II and III). There was no evidence of symmetric transcallosal connectivity influences, since the quadrant mirror-symmetric to the scotoma (II) did not show more pronounced deficits than the diagonal quadrant (III).

In a subsample with quadrantanopia (n = 16) the three intact visual field quadrants were defined according to their location in relation to the scotoma (**Fig. 5**). All three intact quadrants exhibited significantly higher RT than subjects from the normative sample (347.2±4ms). This was true for the adjacent quadrant in the defect ipsilateral hemispace (421.4±17ms; t(1,15) = 4.25, p = 0.001), the adjacent quadrant in the contralateral hemispace (392.5±10ms; t(1,15) = 4.32, p<0.001), and the diagonal quadrant in the contralateral hemispace (391.1±10ms; t(1,15) = 4.19, p<0.001; all tests Bonferroni corrected). Secondly, we found that RT differed in the three intact quadrants as a function of the relative position to the scotoma (F(2,13) = 8.95, p = 0.007). Mean RT in the adjacent quadrant in the same hemispace (421.4±17ms) was significantly longer than in the adjacent quadrant in the contralateral hemispace (392.5±10ms; p = 0.040) or the diagonal quadrant (391.1±10ms; p = 0.012).

## Discussion

The present study demonstrates that visual processing speed, as indicated by reaction time (RT) to simple light stimuli, is impaired in the “intact” visual field of patients with visual system damage. Moreover, two correlates of processing speed in the intact visual field were identified. Firstly, RT in each tested position in the intact visual field is related to the functional state of its “local” surround: the more defective the surround the longer the RT. Secondly, the size of the scotoma matters: the bigger it is, the longer the average intact field RT, which indicates a “global effect” of the scotoma on the visual field. Thus, the local effect accounts for the within-subject variability and topography of the intact field RT, whereas the global effect for inter-subject variability and general level of performance. From this we conclude that the visual system lesion might affect the “intact” visual field in a local (retinotopic) and a global (non-retinotopic) manner. This raises the question as to possible neurophysiological mechanisms underlying these influences.

### Perceptual deficits in the “intact” visual field of patients

Perceptual deficits in the visual field parts believed to be intact were shown in subjects with visual system damage of different etiologies. Firstly, in patients with unilateral visual cortex lesions several perceptual functions such as contrast and contour perception were found to be impaired in the intact (seeing) field processed by the non-lesioned hemisphere [11]-[13]. Moreover, processing speed stands out as one of the main symptoms, since RT in the “intact” visual field of cortically lesioned patients was elevated in comparison to healthy subjects irrespective of the task [11]-[13], [16]-[18]. Likewise, in patients with optic neuritis, temporal processing and motion detection in the intact field were impaired, despite normal static perception [20], [21]. Here we confirm that in a simple detection task, patients with partial visual field loss exhibit processing speed deficits in the visual field area characterized by normal detection abilities. Further, evidence was found for both local and global, but not interhemispheric influences of the scotoma on temporal processing of the intact visual field.

### Local effect of the visual system lesion

The intact field close to the scotoma is characterized by a slower processing speed when compared to the intact sectors with a relatively intact surround (**Fig. 3**). This factor is calculated for each visual stimulus position. This “local scotoma effect” accounts for within-subject variability and determines the topography of the impairment.

What are possible mechanisms of this local effect? Firstly, it has been postulated that in patients with visual field loss attention is not equally distributed across the visual field [4]. Particularly, areas surrounding the scotoma lack attentional reinforcement, as patients might (unintentionally) use visual field areas far from the blind field for everyday vision. This might be reversed by vision restoration training (VRT) combined with attentional cueing [26]. If subjects focus their attention far from the scotoma, processing in the intact field close to the scotoma border might be disturbed. This type of spontaneous attention shift after lesions to the intact field regions resembles the situation of the so called “preferred retinal location” (PRL) observed in age-related macular degeneration subjects (AMD) and might involve similar physiological changes [31]–[33].

There are also several neurophysiological mechanisms that might be of relevance. Firstly, partial optic nerve damage or cortical tissue loss may lead to a reduced physiological signaling power and to a loss of firing synchrony of neuronal assemblies, thus increasing processing time. Secondly, there is an extensive remapping and reorganization of the visual field representation after pre- and post-chiasmatic damage as shown in animal model studies [34]–[37], as well as behavioral [38] and fMRI studies in patients [31]–[33], [39]. Such receptive field reorganization is believed to be mediated by mechanisms involving plasticity of intrinsic cortical horizontal connections [40]. Importantly, receptive fields modifications are found not only within the damaged tissue or lesion projection zone, but they also occur in the area adjacent to the scotoma but not directly affected by the lesion [35], [37]. In this context a study by Dilks et al. [38] showed that shapes presented in the intact quadrant adjacent to the scotoma (in the same hemispace) are distorted and often elongated toward and into the blind field. Presumably, receptive fields shift shown in animal models might produce this kind of spatial distortions. This is in line with our analysis of RT in quadrantanopia patients, revealing that the quadrant adjacent to the scotoma is most impaired in terms of processing speed (**Fig. 5**). Thirdly, receptive fields not only change their location but also become larger [34], [35]. It is still unclear how these changes affect perception. While an enlarged receptive field implies pooling of signals from larger regions of the visual field, thus increasing the probability of responding to stimuli, it may also introduce more noise. Future studies need to show if or when receptive fields plasticity is adaptive or maladaptive.

### Global effect of visual system lesions

Besides local influences we also found evidence for a global (non-retinotopic) influence of the scotoma on the intact visual field. Specifically, a larger scotoma was associated with a greater RT deficit in the intact visual field (**Fig. 4**) suggesting that the amount of spared cortical tissue is related to the efficacy of intact field processing [11]. This cannot be explained by local influences alone, because when the correlation analysis of scotoma size and intact field RT was repeated after RT normalization for the functional state of the surround, the correlation remained significant. Secondly, both groups of patients exhibited slower RT than healthy subjects when only intact sectors with completely intact surrounds were taken into account. Finally, a global effect was further substantiated by analysis of RT in quadrantanopia subjects. All three intact quadrants were impaired in comparison to healthy subjects, including the quadrant diagonal to the scotoma which is not influenced by any local interactions (see: **Fig. 5**).

Given the extensive connectivity of the visual system [41] it is hardly surprising that a lesion or deafferentation of the primary visual cortex has a non-specific “global effect” on visual processing. Alterations in down-stream, higher cortical areas, not directly affected by the lesion, were already demonstrated by gene expression studies [42] and single-unit recordings in animal models [43], [44], as well as MEG [45] and fMRI studies in humans [20], [46]–[52]. This is in line with findings from motor [53], [54] and attentional systems [55] where lesion-induced network modifications have been increasingly recognized and appreciated. However, the behavioral correlates of network alterations are still poorly understood in all systems. Our findings point towards visual processing speed deficits as a possible, non-specific consequence of the visual system lesion that might be the result of widespread neuronal network changes.

### No evidence for interhemispheric effects of unilateral visual cortex lesion

Further, because of the extensive interhemispheric connectivity in the visual system [56], [57], areas within the intact hemisphere that are mirror-symmetrically located to the lesion are likely to suffer functional deficits because their transcallosal input is lost. After a unilateral visual cortex lesion the activity patterns of the seemingly “unaffected” hemisphere were found to be altered [50], [52], [58]. In addition, interhemispheric anatomical and functional connectivity undergoes modifications, as shown by two case studies [59], [60]. Connectivity changes following visual cortex damage [61] and temporal inactivity induced by transcranial magnetic stimulation in healthy subjects [62] which interfered with processing in the “intact” visual cortex demonstrate that unilateral visual cortex lesions or deactivations might affect processing on the “intact” side. However, despite ample evidence emphasizing the importance of interhemispheric connection in perception [63], [64], we did not find any effect of the missing symmetric interhemispheric connectivity in quadrantanopia subjects with our simple detection tasks: although all quadrants were impaired in comparison to the non-injured control sample, the quadrant opposite of the lesion had a similar RT impairment as the quadrant diagonal to the lesion (**Fig. 5**). Only the intact quadrant processed by the lesioned hemisphere was clearly more impaired than the other two intact quadrants. Therefore the question arises, whether the effect of disturbed transcallosal connectivity might only be measurable if more complex tasks are used, such as perceptual grouping and gestalt integration [12], [13].

### Clinical relevance of the intact field perceptual deficits

We propose that the intact visual field dysfunction, here temporal processing, has an impact on overall subjective vision in patients. Since the scotoma size does not always match well with the subjective vision loss, as measured by vision-related quality of life questionnaires [4], [22], other factors than scotoma size may contribute to the patients` subjective estimates of visual functions and the perceptual impairment in the seeing field may offer a possible explanation for this (for a discussion of the “mismatch-problem”, see [4]).

The question arises why so little is known about intact field deficits. We believe the main reason is that patients with visual system damage are usually diagnosed with standard perimetry using simple detection tasks which are “blind” with respect to higher perceptual and cognitive functions (e.g. feature binding, attentional selection), or dynamic perception (e.g. motion detection). Only in single clinical studies RT to perimetric stimuli was analyzed, showing high variability of RT among patients [65]. Therefore, to gain a better understanding of the subjective element of blindness, visual function beyond simple detection in a standard vision exam should be assessed to fully appreciate the objective and subjective vision loss [21].

### Conclusions

By analyzing RT in a simple perimetric light detection task it was shown that patients with visual field loss suffer processing speed deficits in visual field areas previously considered to be intact. In both groups, pre- and post-chiasmatic, visual system lesions have local (retinotopic) and global (non-retinotopic) effects on intact field processing speed. The local effect is shown by slower processing of visual field nearby the scotoma, while the global effect is indicated by an association between larger scotoma with longer RT in the intact visual field. Thus, visual system lesions have more far-reaching consequences for visual functions than previously expected, affecting the presumed “intact” visual field as well.

The slowing down of intact field temporal processing might explain why patients complain that visual scenes in the world outside their homes move “too fast”, making them feel insecure in busy environments. Judging the patients vision loss based solely on detection accuracy in perimetry underestimates the subjective visual impairment. This has also implication for neurovisual rehabilitation methods such as vision restoration training [25], [27], non-invasive current stimulation [66], [67], or both combined [68], [69]. They might be used to also improve these less obvious deficits in the intact visual field sector. By studying subtle perceptual deficits in partial blindness we may better understand the subjective representation of visual capabilities of patients but also improve therapeutic approaches aiming at restoration of vision and increasing visual competency during rehabilitation.
